# Anti-IL-17 Inhibits PINK1/Parkin Autophagy and M1 Macrophage Polarization in Rheumatic Heart Disease

**DOI:** 10.1007/s10753-024-02094-3

**Published:** 2024-07-08

**Authors:** Ling Bai, Yuan Li, Chuanghong Lu, Yiping Yang, Jie Zhang, Zirong Lu, Keke Huang, Shenglin Xian, Xi Yang, Na Na, Feng Huang, Zhiyu Zeng

**Affiliations:** 1 Department of Cardiology, The First Affiliated Hospital of Guangxi Medical University, Shuang Yong Road 6, Nanning, 530021 Guangxi China; 2 Guangxi Key Laboratory Base of Precision Medicine in Cardio-Cerebrovascular Diseases Control and Prevention, Guangxi Clinical Research Center for Cardio-Cerebrovascular Diseases, Nanning, Guangxi China; 3 Department of Research, Guangxi Medical University Cancer Hospital, Nanning, Guangxi China; 4 Emergency Office, Nanning Center for Disease Control and Prevention, Nanning , Guangxi China; 5 Department of Endocrinology, The First Affiliated Hospital of Guangxi Medical University, Nanning, Guangxi China; 6 Department of Neuroscience, The Scripps Research Institute, La Jolla, USA

**Keywords:** il-17, macrophage, autophagy, polarization, pink1/parkin, rheumatic heart disease

## Abstract

Rheumatic heart disease (RHD) is an important and preventable cause of cardiovascular death and disability, but the lack of clarity about its exact mechanisms makes it more difficult to find alternative methods or prevention and treatment. We previously demonstrated that increased IL-17 expression plays a crucial role in the development of RHD-related valvular inflammatory injury. Macrophage autophagy/polarization may be a pro-survival strategy in the initiation and resolution of the inflammatory process. This study investigated the mechanism by which IL-17 regulates autophagy/polarization activation in macrophages. A RHD rat model was generated, and the effects of anti-IL-17 and 3-methyladenine (3-MA) were analyzed. The molecular mechanisms underlying IL-17-induced macrophage autophagy/polarization were investigated via in vitro experiments. In our established RHD rat model, the activation of the macrophage PINK1/Parkin autophagic pathway in valve tissue was accompanied by M1 macrophage infiltration, and anti-IL-17 treatment inhibited autophagy and reversed macrophage inflammatory infiltration, thereby attenuating endothelial–mesenchymal transition (EndMT) in the valve tissue. The efficacy of 3-MA treatment was similar to that of anti-IL-17 treatment. Furthermore, in THP-1 cells, the pharmacological promotion of autophagy by IL-17 induced M1-type polarization, whereas the inhibition of autophagy by 3-MA reversed this process. Mechanistically, silencing PINK1 in THP-1 blocked autophagic flux. Moreover, IL-17-induced M1-polarized macrophages promoted EndMT in HUVECs. This study revealed that IL-17 plays an important role in EndMT in RHD via the PINK1/Parkin autophagic pathway and macrophage polarization, providing a potential therapeutic target.

**Supplementary Information:**

The online version contains supplementary material available at 10.1007/s10753-024-02094-3.

## INTRODUCTION

Rheumatic heart disease (RHD) is an autoimmune condition that develops due to recurring bouts of acute rheumatic fever following infection with group A hemolytic streptococci (GAS) [1]. RHD is a prevalent acquired illness among children in developing countries, posing a substantial burden on morbidity and mortality rates in low- and middle-income nations [2, 3]. In these regions, the limited availability of antibiotics and complex surgical interventions present considerable challenges in effectively halting the progression of RHD [4, 5]. Additionally, the elusive understanding of the precise mechanism by which RHD causes damage to heart valves further complicates the search for alternative methods of prevention and treatment. Our previous studies revealed that the partial transformation of endothelial cells to a mesenchymal phenotype in cardiac valves, a process known as endothelial-mesenchymal transition (EndMT), is a key player in the development of RHD-related valvular fibrosis [6]. In chronic inflammation, the induction of EndMT processes by inflammatory mediators, including interleukins, plays an important role in disease progression [7].

Macrophage heterogeneity (M1/M2 polarization) is essential for cardiovascular health, with "proinflammatory and lethal" as well as "anti-inflammatory and reparative" functions in the body's microenvironment in predominantly inflammatory disease states [8–11]. Increased recruitment and infiltration of M1-type macrophages in tissues can drive the associated inflammatory response, leading to fibrosis [12, 13]. However, whether macrophage polarization is involved in RHD heart valve EndMT is unclear. Autophagy plays an important role in macrophage polarization and can participate in the inflammatory response by regulating macrophage polarization [14–17]. Autophagy enables cells to continue to function normally by degrading and recycling proteins and damaged organelles in lysosomes [18, 19]. Excessive autophagy has been associated with valve diseases, leading to inflammatory cell recruitment and calcification [20–22]. Although the role of autophagy in macrophage polarization and inflammation has been extensively studied, its specific role in RHD and its interaction with macrophage polarization in this context have not been well characterized. The PTEN-induced putative kinase protein 1 (PINK1)/Parkin pathway is a well-known autophagic pathway [23]. Recent evidence suggests that PINK1-mediated autophagy may exert a protective effect on inflammatory responses [24].

The expression of IL-17 in tissues has been found to be positively correlated with the severity of the inflammatory response in autoimmune diseases, including RHD [25, 26]. A previous study provided compelling evidence that peripheral blood Th17/Treg ratios and serum IL-17 concentrations are markedly greater in RHD patients than in healthy individuals [27]. Additionally, we revealed a significant increase in IL-17 expression in the valve tissues and serum of both acute and chronic RHD model rats [28, 29]. These findings suggest that persistently activated IL-17 plays an important regulatory role in the development of inflammatory injury in RHD patients, but the specific molecular regulatory mechanism involved remains unknown. Indeed, studies have demonstrated a close association between IL-17-mediated immune-inflammatory responses and macrophages [30, 31]. To further explore the underlying mechanisms, we used anti-IL-17 and the autophagy inhibitor 3-methyladenine (3-MA) in a rat model of RHD, and performed in vitro studies using THP-1 cells and HUVECs. In this study, we demonstrated that M1 polarization of macrophages was related to the activation of the PINK1/Parkin autophagy signaling pathway via IL-17, which led to EndMT. Overall, our study reveals a new theoretical basis and potential intervention targets for preventing and treating inflammatory damage in RHD patients.

## MATERIALS AND METHODS

### Experimental Animals

Eight-week-old adult female Lewis rats weighing between 160–180 g were procured from Beijing Vital River Laboratory Animal Technology Co., Ltd. The rats were housed in a specific pathogen-free (SPF) animal laboratory at the Animal Experiment Center of Guangxi Medical University and maintained at a temperature of 23 ± 2 °C, with a 12-h light/dark cycle. They were provided with free access to water and feed, and were allowed unrestricted movement within their cages. Prior to the commencement of the experiments, the rats were acclimated to the experimental environment for a period of 5 days. The procedures employed in this investigation adhered to the ethical standards for the care and utilization of laboratory animals, and received approval from the Experimental Animal Welfare and Ethics Committee of Guangxi Medical University (202303009).

### Antigen Preparation

GAS (American Type Culture Collection [ATCC]) was cultured in brain heart infusion medium (Guangdong Huankai Microbial Sci. & Tech. Co., Ltd.) at 37 °C for 24 h, followed by washing with normal saline (NS). After harvesting, the GAS was incubated in 10% neutral formalin for 12 h. The inactivated GAS was then washed and resuspended in NS, and the density was adjusted to 4.0 × 10^11^ colony forming units (CFU)/ml. Ultrasound was used to emulsify the suspensions and prepare the antigens (Sonics & Materials, Inc.).

### Groups and Treatments

The rats were randomly divided into 5 groups: the control group, RHD group, anti-IL-17 treatment group, 3-MA treatment group, and anti-IL-17 + 3-MA treatment group. It took a total of 8 weeks to establish the RHD animal experimental model, following the procedure reported previously [6, 28]. First, a total volume of 100 µl, containing inactivated GAS (4.0 × 10^11^ CFU/ml) and complete Freund's adjuvant (CFA, Sigma‑Aldrich) at a 1:1 ratio, was injected into one of the hindfoot pads of the rats. After one week, 500 µl of the same solution was injected subcutaneously intraperitoneally at weekly intervals for 4 weeks. During the final 4 weeks, subcutaneous intraperitoneal injections were administered weekly, with the injection volume was adjusted to 500 µl of inactivated GAS (4.0 × 10^11^ CFU/ml). Anti-IL-17 treatment commenced from the second week of RHD modeling and continued for a total of 7 week. The rats were injected intravenously (i.v.) twice weekly of 0.1 mg/kg (rat body weight) anti-IL-17 antibody (Thermo Fisher Scientific), which was dissolved in PBS. 3-MA (15 mg/kg, GLPBIO) in PBS was administered intraperitoneally starting one week after RHD induction and continued twice weekly for 7 weeks in the 3-MA treated group. The anti-IL-17 + 3-MA group received a combined treatment. The control group underwent the same injection protocol as the RHD group, but with an equal volume of NS as the injection solution (Fig. S1).

### Animal Sacrifice

After the treatments were completed, 2 ml of blood was drawn from the rat's tail vein, followed by an intraperitoneal injection of pentobarbital sodium (150 mg/kg). Death was confirmed if no signs of respiration or heartbeat were observed for > 5 min. A body weight loss exceeding 15% and a decreased ability to consume food and water were considered humane endpoints.

### Sample Preparation

Mitral valve specimens were quickly excised from the sacrificed rats, flash-frozen in liquid nitrogen, and stored at -80 °C for subsequent experimental analysis. To minimize the impact of temperature fluctuations on the samples, all specimens were kept in a refrigerator at a constant temperature of -80 °C until the experiment was conducted. One section from each mitral valve specimen was fixed in 4% paraformaldehyde and embedded in paraffin.

### Histochemistry

After 12 h in 4% paraformaldehyde, the blocks were dehydrated and then immediately placed in melted paraffin wax, keeping it warm until the tissue was completely immersed. All tissue blocks were serially sectioned at a thickness of 5 µm for hematoxylin and eosin (H&E, Servicebio) and Sirius Red staining (Servicebio). H&E staining images were acquired using a BX43 light microscope (Olympus Corporation). Sirius Red staining images (polarized light) were acquired using a BX43 confocal microscope (Olympus Corporation).

### Immunohistochemistry

Immunohistochemical analysis was conducted using purified polyclonal antibodies against rat IL-17 (1:100, DEVELOP), LC3 (1:200, Abcam), CD68 (1:200, Servicebio), CD206 (1:500, Servicebio), CD31 (1:2000, Abcam) and vimentin (1:200, Abcam). Paraffin-embedded sections were incubated in a 3% hydrogen peroxide solution at room temperature in the dark for 25 min. The sections were blocked with 3% BSA (Servicebio) for 30 min at room temperature, followed by incubation with the respective primary antibodies mentioned above for 12 h at 4 °C, followed by 50 min of incubation with the horseradish peroxidase (HRP)‑conjugated anti‑rabbit (1:200, Servicebio) secondary antibody at room temperature. The results were interpreted under a BX43 light microscope (Olympus Corporation); positive expression was indicated by a brownish-yellow color, and quantitative analysis was performed using Image-Pro Plus 6.0.

### Cell Culture

THP-1 cells (Procell) were cultured in RPMI-1640 medium (Procell) supplemented with 10% fetal bovine serum (FBS) and 1% penicillin–streptomycin solution (P/S) at 37 °C under 5% CO_2_. Next, 5 × 10^5^ cells/mL THP-1 cells were seeded in 6-well plates and differentiated into macrophages by 48 h of incubation with 100 ng/mL phorbol-12-myristate-13-acetate (PMA, Sigma-Aldrich). Following a 2-h pretreatment with or without 3-MA (10 mM, Sigma-Aldrich), cells were stimulated with or without IL-17 (50 ng/ml, PeproTech) for 24 h. The resulting supernatants were then collected after the respective interventions and stored at -20 °C as conditioned medium (CM) for use as backup samples.

HUVECs (ATCC) were cultured in ECM (Sciencell) supplemented with 5% FBS, 1% endothelial cell growth supplement and 1% P/S at 37 °C and 5% CO_2_ in a humidified atmosphere. The CM from macrophages in different activation states, as described above, was mixed with complete ECM at a 1:1 ratio to treat HUVECs for 24 h, with the concentration adjusted to 2 × 10^5^ cells/mL.

### Electron Microscopy

The cells were fixed with electron microscopy fixative (Servicebio) for 2 h at 4 °C. After washing in 0.1 M phosphate buffer, they were further fixed with 1% osmium tetroxide for 2 h at 4 °C, dehydrated using ethyl alcohol solutions, and then exchanged with acetone overnight before being embedded in Epon 812. Ultrathin Sects. (60–80 nm) were obtained by using an ultramicrotome, and images were captured with a transmission electron microscope (HITACHI).

### Immunofluorescence

Paraffin-embedded sections were sealed with 3% BSA (Servicebio) at room temperature for 30 min. The sections were then incubated separately with purified anti-rat CD68 antibody (1:2000, Servicebio) and LC3 antibody (1:200, Abcam) at 4 °C for 12 h. Then the corresponding HRP-conjugated secondary antibodies were added, and the sections were incubated at room temperature for 50 min. After incubation with DAPI staining solution at room temperature for 10 min, the sections were observed under a BX43 optical microscope (Olympus Corporation).

The cells were fixed with 4% paraformaldehyde for 15 min, gently washed 3 times with PBS, and then permeabilized with PBS containing 0.5% Triton X-100 for 15 min at room temperature. The cells were blocked in immunofluorescence blocking solution (Beyotime) for 30 min at room temperature and incubated with an anti-LC3 antibody (1:20, Abcam) at 4 °C overnight. On the following day, the cells were incubated with goat anti-rabbit Alexa Fluor® 594IgG or 488IgG secondary antibodies (1:1000, Abcam) for 1 h in the dark. The cell nuclei were then stained with DAPI for 5 min in the dark and observed under a BX43 light microscope (Olympus Corporation).

### siRNA Transfection

siRNA was synthesized by HANBIO in Shanghai, China. THP-1 cells were transfected with 100 nM siRNA using Lipofectamine 3000 (Thermo Fisher Scientific) following the manufacturer's protocol. The PINK1 siRNA sequence is shown in Table S1.

### Reverse Transcription‑quantitative PCR (RT‑qPCR)

Total RNA was extracted from heart valve tissue or cultured cells using RNAiso Plus (Takara) according to the manufacturer's recommended procedure. RNA was reverse transcribed into cDNA using a reagent kit (Takara), and the entire reverse transcription process was performed following the manufacturer's instructions. RT‑qPCR was conducted using TB Green Premix Ex Taq II (Takara) on a real-time PCR system (Bio-Rad). The final results are expressed as the fold difference relative to the endogenous housekeeping gene for each mRNA, calculated using the 2^‑ΔΔct^ method. The sequences of the primers used are listed in Table S2.

### Enzyme‑linked Immunosorbent Assay (ELISA)

The concentrations of IL-17, IL-1β, IL-6, TNF-α, TGF-β, and IL-10 in the supernatants of cultured cells or serum from RHD model rats were measured using ELISA kits (Servicebio) following to the manufacturer’s procedures.

### Western Blotting (WB)

Heart valve tissue and cultured cells were lysed in a mixture of ice-cold RIPA buffer (Solarbio) and phenylmethylsulfonyl fluoride (PMSF, Solarbio) at a ratio of 1:100 for 1 h.The protein concentration was determined using BCA assays (Beyotime). Protein samples were separated by 10% or 15% SDS-PAGE (New Cell & Molecular Biotech) and transferred to a PVDF membrane. The following antibodies were used for WB analysis: anti-GAPDH (1:10000, Abcam), anti-LC3 (1:2000, Abcam), anti-Beclin1 (1:1000, Abcam), anti-p62 (1:10000, Abcam), anti-PINK1 (1:2500, Cloud-Clone Corp), anti-Parkin (1:2000, Abcam), anti-α-SMA (1:10000, Abcam), anti-vimentin (1:1000, Abcam), and anti-VE-cadherin (1:1000, Abcam).

### Flow Cytometry

The cells were washed three times with precooled staining buffer (BD Pharmingen), incubated with fixation/permeabilization solution (BD Pharmingen) for 20 min, washed twice with Perm/Wash buffer, and stained with CD86-FITC (BD Pharmingen), CD80-PE-CY7 (BD Pharmingen), CD206-PE (BD Pharmingen), and CD163-Percpcy5.5 (BioLegend) for 30 min at 4 °C in the dark. After the final washing step, the labeled cells were resuspended in 500 µL stain buffer and analyzed on a flow cytometer using CytExpert software.

### Statistical Analysis

The data are expressed as means ± standard deviations (SDs). Two-tailed unpaired Student’s *t-*tests were used to compare the experimental and control groups. One-way ANOVA was performed to analyze differences between multiple groups. All statistical analyses were performed using SPSS software version 20.0 for Windows (SPSS, Inc., Chicago, IL, USA), and GraphPad Prism 9.0 software was used to plot the graphs. A *p*-value < 0.05 was considered to indicate statistical significance.

## RESULTS

### Anti-IL-17 Treatment Effectively Attenuates EndMT and Fibrosis Triggered by Macrophage M1 Polarization

**Fig. 1:**
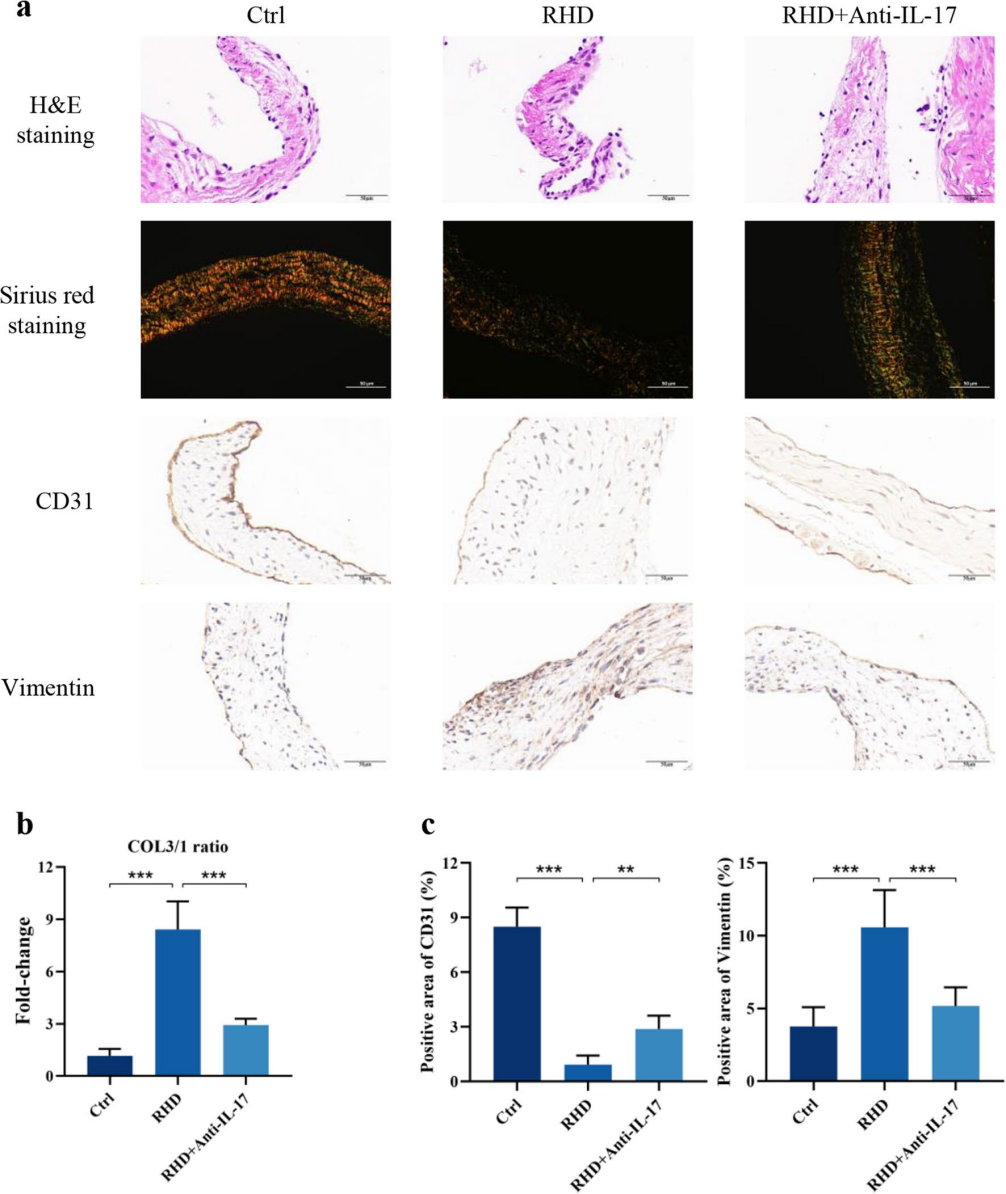
Anti-IL-17 treatment effectively attenuates EndMT and fibrosis in RHD rats. **a** H&E staining, sirius red staining (COL1 fibers show tightly arranged yellow and red, and COL3 fibers show loosely arranged green), and immunohistochemical staining for CD31 and Vimentin of rat mitral valve tissue. Scale bar = 50 µm. **b** COL3/1 ratio indicates decreased fibrosis in mitral valve tissue of RHD rats after anti-IL-17 treatment (*n* = 6). **c** Anti-il-17 reversed the significant decrease in CD31-positive areas and the significant increase in Vimentin-positive areas in the RHD group (*n* = 6). ^*^P < 0.05, ^**^P < 0.01, ^***^P < 0.001.

H&E staining showed diffuse infiltration of inflammatory cells in the mitral valve of RHD model rats. Although there were no differences in body weight measurements between the groups (Fig. S2), remission of the inflammatory response reached significance during the period of treatment with anti-IL-17 (Fig. 1a). Sirius red staining distinguishes the type of collagen fibers, and previous studies have demonstrated that a significant increase in the COL3/1 ratio can be used to determine the onset of valvular fibrosis [28]. The results showed that the COL3/1 ratio was significantly higher in the RHD group than in the control group. Compared with that in the RHD group, the COL3/1 ratio in the anti-IL-17 group was significantly decreased (Fig. 1a, b).

**Fig. 2:**
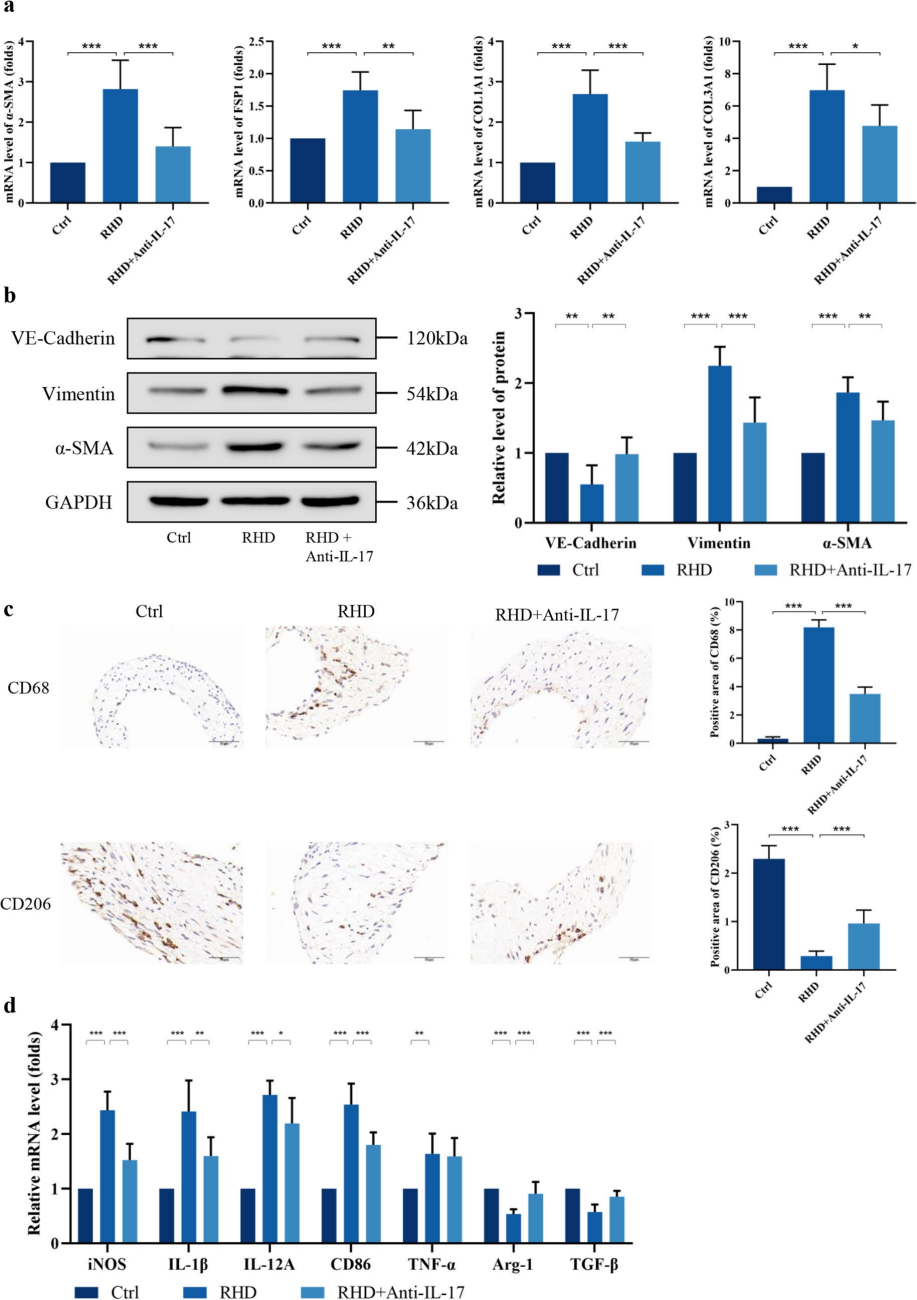
Anti-IL-17 treatment effectively attenuates EndMT and fibrosis triggered by macrophage M1 polarization in rats. IL-17 reduces mRNA (**a**) and protein (**b**) expression levels of EndMT and fibrosis-related factors in RHD valve tissues (*n* = 6). **c** Immunohistochemical staining for CD68 and CD206 (*n* = 6). Scale bar = 50 µm. **d** IL-17 reduces the proportion of M1-Type macrophages in RHD valve tissues (*n* = 6). ^*^P < 0.05, ^**^P < 0.01, ^***^P < 0.001.

Immunohistochemistry showed that the area of vimentin-positive cells in the RHD groups was significantly greater than that in the control group. Anti-IL-17 treatment also significantly attenuated the vimentin-positive areas in the valve tissues. In addition, the trend of the CD31-positive area in the valve tissues of rats in each experimental group was opposite to that of the vimentin-positive area (Fig. 1a, c). In model RHD rat valve tissues, elevated expression of α-SMA, fibroblast‑specific protein 1 (FSP1), collagen type I α1 (COL1A1), and collagen type III α1 (COL3A1) mRNA was measured by RT‑qPCR. The expression levels of α-SMA, FSP1, COL1A1, and COL3A1 was considerably lower in the anti-IL-17 group than in the RHD group, which indicated a decrease in valve fibrosis (Fig. 2a). Later, we used WB to examine the protein expression of EndMT-related genes in the mitral valve tissues of the rats. Consistent with the mRNA results, the WB results showed that the protein expression of α-SMA in the RHD group was markedly higher than that in the control group and significantly lower in the anti-IL-17 group than in the RHD group. Among other EndMT-related genes, VE-cadherin protein expression was significantly lower in RHD valve tissues than in control tissues, while vimentin protein expression was significantly higher in RHD valve tissues than in control tissues. After anti-IL-17 treatment, the protein expression of VE-cadherin and vimentin tended to increase or decrease, respectively, and the differences were statistically significant (Fig. 2b). Together, these data confirm the involvement of EndMT in the pathological process of acute RHD mitral valve lesions and demonstrate that anti-IL-17 therapy effectively reduces inflammatory infiltration and fibrosis of the valve.

Immunohistochemical results revealed that the CD68-positive areas were significantly increased in the RHD group and significantly decreased in the anti-IL-17 group, whereas the CD206-positive areas showed the opposite trend (Fig. 2c). The levels of M1/M2-related cytokines were measured by RT-qPCR in the valve tissues of RHD model rats, and the mRNA levels of iNOS, IL-1β, IL-12A, and CD86 were found to be significantly higher in the RHD group than in the control group. The levels of these cytokines were significantly decreased after anti-IL-17 treatment. However, no decreasing trend in TNF-α was detected. The expression of Arg-1 and TGF-β in the RHD group was significantly lower than that in the control group and tended to rebound after anti-IL-17 treatment (Fig. 2d). This finding suggested that inflammatory injury in RHD valves is associated with macrophage M1 polarization, and IL-17 may regulate this polarization process.

**Fig. 3:**
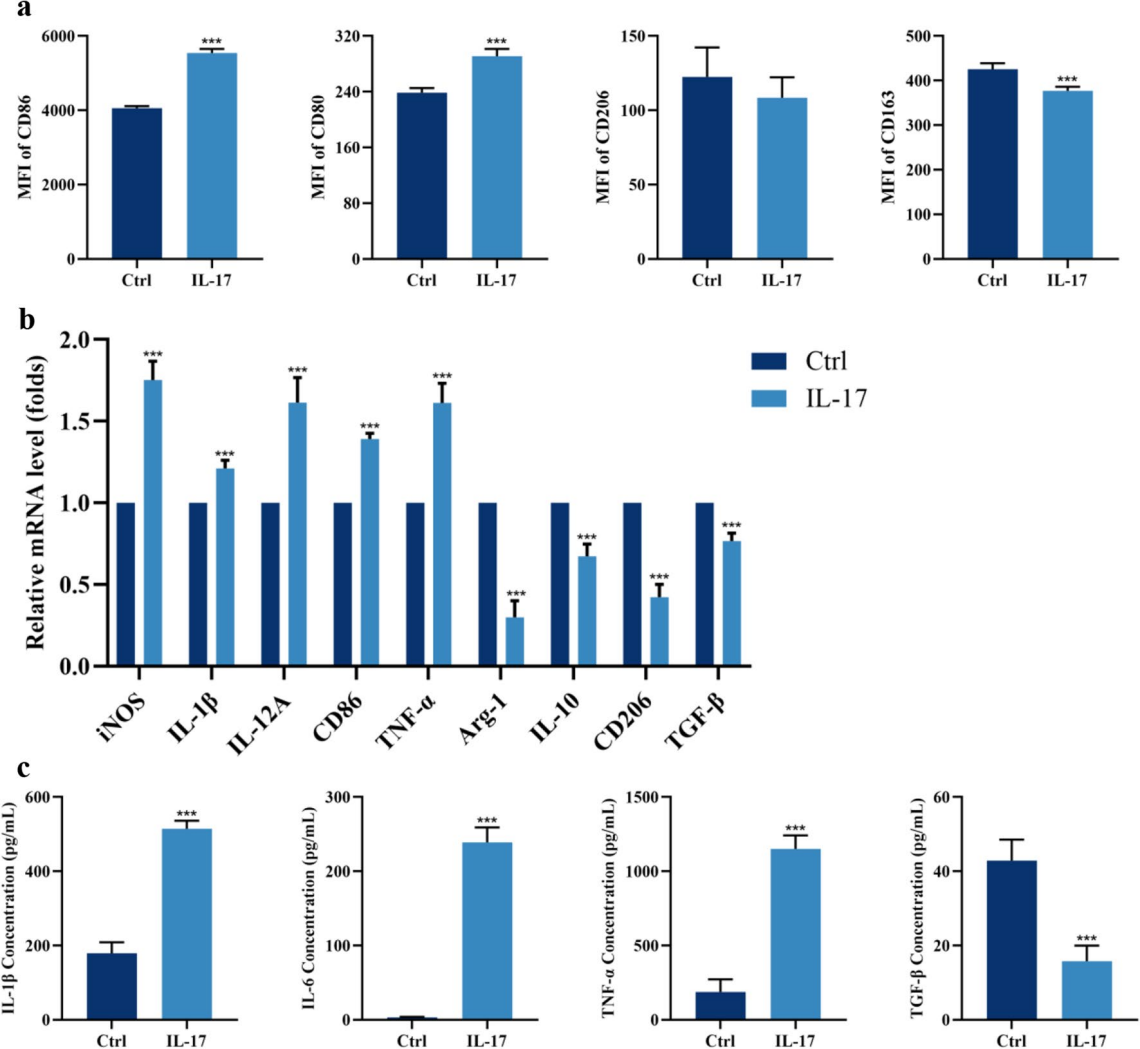
Exposure to exogenous IL-17 enhances polarization of M1 macrophages. **a** IL-17 increases the MFI of M1-type markers in THP-1 macrophages (*n* = 5). **b** IL-17 increases the mRNA expression level of M1-macrophage-associated factors and decreases the mRNA expression level of M2-associated factors (*n* = 5). **c** Elevated levels of pro-inflammatory cytokines in THP-1 medium supernatants after IL-17 intervention (*n* = 5). ^*^P < 0.05, ^**^P < 0.01, ^***^P < 0.001.

**Fig. 4:**
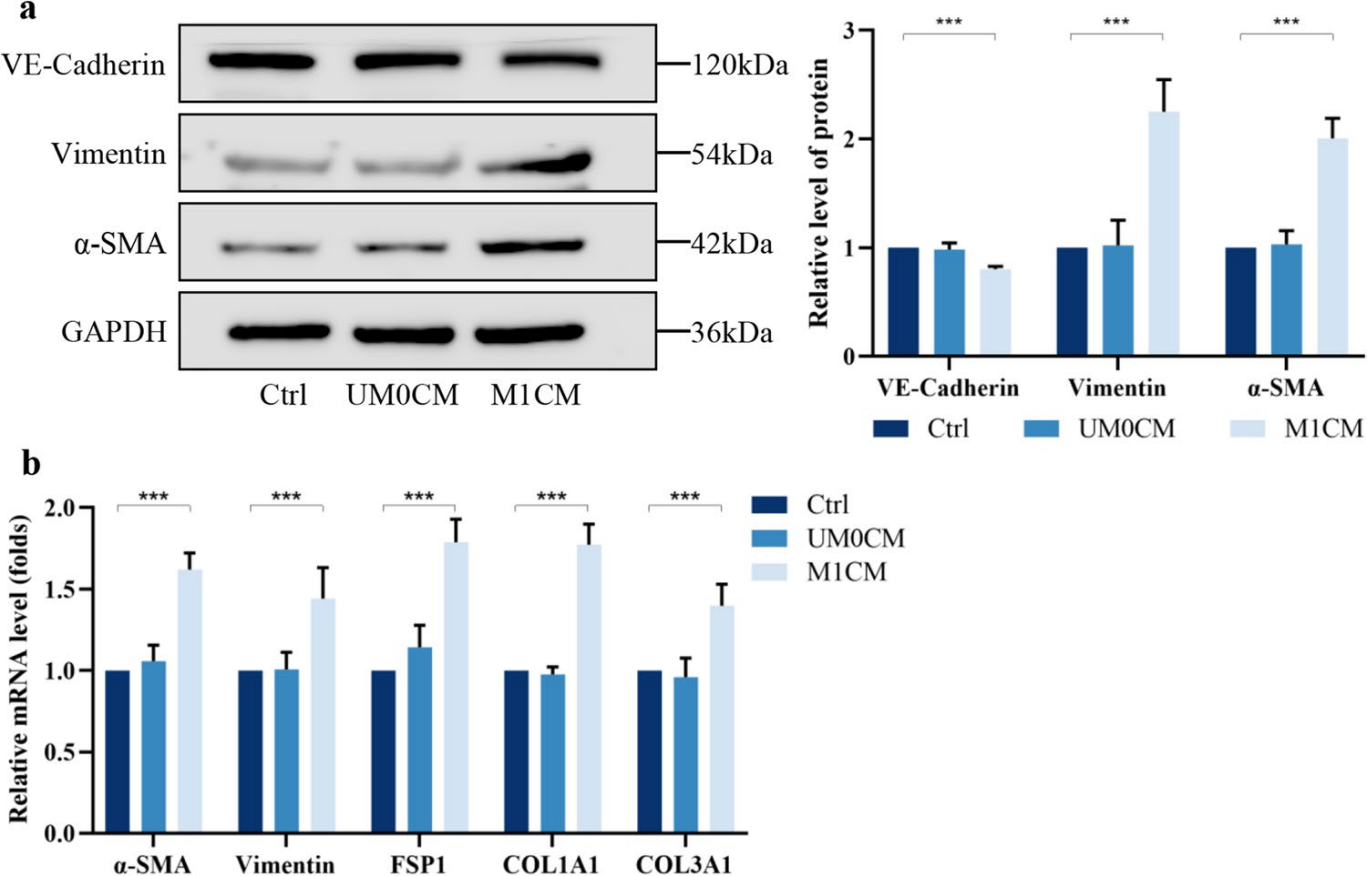
Co-culture with HUVECs using M1CM induces EndMT. Co-culture with IL-17-interacted macrophages (M1-macrophages) elevates EndMT related protein (**a**) and mRNA (**b**) expression levels in HUVECs (*n* = 5). ^*^P < 0.05, ^**^P < 0.01, ^***^P < 0.001.

Next, we explored macrophage polarization in the presence or absence of IL-17. The flow cytometry results showed that macrophages treated with IL-17 expressed higher mean fluorescence intensities (MFIs) of CD80-PE-CY7 and CD86-FITC and lower levels of CD163-Percpcy5.5 (Fig. 3a). We also assessed the mRNA expression of known M1 and M2 macrophage markers in THP-1 cells after IL-17 intervention. IL-17 promoted M1 polarization, as determined by the detection of M1 (iNOS, IL-1β, IL-12A, CD86, TNF-α) and M2 (Arg-1, IL-10, CD206, TGF-β) markers (Fig. 3b). Exposure to exogenous IL-17 enhanced M1 macrophage polarization, as evidenced by increased secretion of the proinflammatory cytokines IL-1β, IL-6, and TNF-α, and decreased secretion of the inhibitory cytokine TGF-β (Fig. 3c). To further investigate the effects of different polarization states of macrophages on HUVECs, we selected supernatants from undifferentiated macrophages (UM0) and macrophages after IL-17 intervention (M1-type macrophages) for co-culture with HUVECs. The results showed that M1CM decreased the protein abundance of VE-cadherin but increased the protein abundance of vimentin and α-SMA (Fig. 4a). The mRNA levels of EndMT-related and fibrosis-related genes (FSP1, COL1A1, and COL3A1) were also increased after the co-culture of HUVECs with M1CM (Fig. 4b). These results suggest that IL-17 induces altered macrophage M1 polarization, which in turn leads to EndMT in HUVECs, and that anti-IL-17 treatment effectively attenuates fibrosis triggered by inflammatory damage to the valves.

### The anti-IL-17-mediated Inhibition of Macrophage Inflammatory Infiltration in RHD Valves is Mediated by Reduced Autophagy Activation

Immunohistochemistry and RT-qPCR revealed a significant increase in IL-17 expression in the RHD group compared to the control group and a significant decrease in IL-17 expression in the anti-IL-17 group compared to the RHD group. The 3-MA group showed the same trend as the anti-IL-17 group. Compared with 3-MA monotherapy, dual therapy was more effective at reducing IL-17 expression (Fig. S3a, b). The results of the rat serum ELISA showed that the IL-17 level in the RHD group was significantly higher than that in the control group. The level of IL-17 in the anti-IL-17 group was significantly lower than that in the RHD group, and the change in the 3-MA group was the same as that in the anti-IL-17 group (Fig. S3c).

**Fig. 5:**
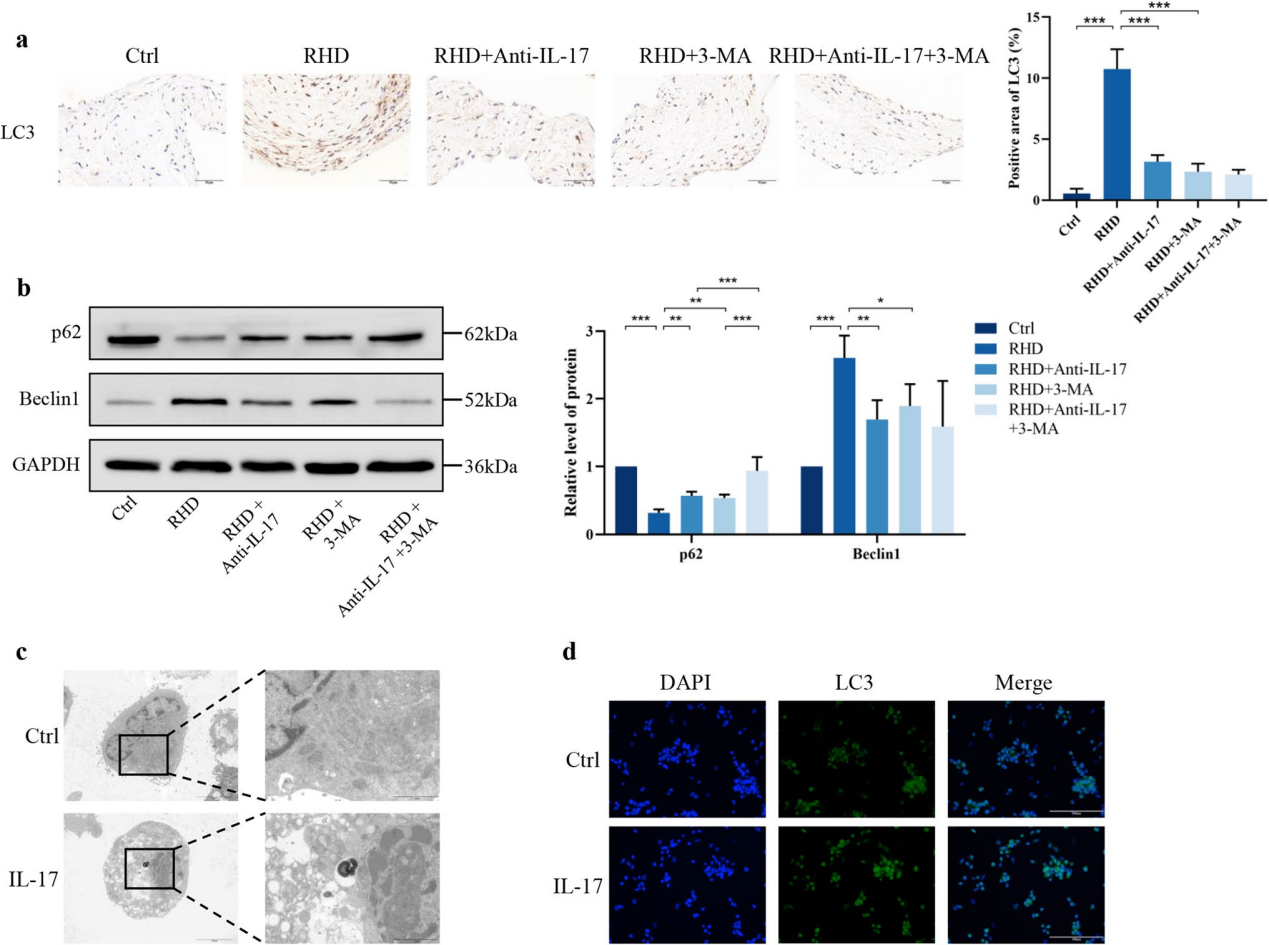
The expression level of IL-17 correlates with autophagic activity. **a** Immunohistochemical staining for LC3 of rat mitral valve tissues (*n* = 6). Scale bar = 50 µm. **b** WB analysis for macrophage autophagy related protein levels (*n* = 6). **c** Electron microscopy images of autophagosomes in vitro. Scale bar = 5.0 µm. **d** Immunofluorescence staining for LC3 in vitro. Scale bar = 200 µm. ^*^P < 0.05, ^**^P < 0.01, ^***^P < 0.001.

In immunofluorescence staining, red fluorescence indicates LC3 protein labeling, green fluorescence indicates CD68 (macrophage marker) protein labeling, and the overlapping position of fluorescent markers indicates yellow fluorescence. Our results showed the colocalization of LC3 and CD68 in the mitral valve tissue of RHD model rats, which indicated that macrophage autophagy was activated (Fig. S4). Immunofluorescence revealed a significant increase in the LC3-positive area in the RHD group and a significant decrease in the anti-IL-17 and 3-MA groups, but no synergistic effect of the combination treatment was detected (Fig. 5a). To further investigate the role of autophagy in mitral valve pathology in RHD, we used 3-MA, a typical autophagy inhibitor, and anti-IL-17 in our well-established RHD rat model. We examined the expression of the autophagy-associated proteins Beclin1 and p62 and found that Beclin1 expression was increased and p62 expression was decreased in the RHD group compared to the control group, suggesting that autophagy was activated. In contrast, anti-IL-17 and 3-MA monotherapy significantly decreased the expression of Beclin1 and restored the expression of p62 compared to those in the RHD group. In addition, dual blockade significantly increased p62 expression more than anti-IL-17 and 3-MA monotherapy (Fig. 5b). Thus, macrophage autophagy is involved in the pathogenesis of RHD, and anti-IL-17 treatment inhibits autophagy activation.

To investigate the effects of IL‑17 on macrophage function, THP-1 cells were cultured in medium supplemented with or without IL‑17. Electron microscopy revealed that after IL-17 treatment, the cell membrane was slightly disrupted locally, the mitochondria were damaged, the cristae were obviously disrupted, and autophagic vesicles were present in the cytosol (Fig. 5c). WB analysis revealed that the expression of Beclin1 and LC3-II was upregulated and that of p62 was downregulated after the addition of IL‑17, suggesting that autophagosome formation was enhanced (Fig. S5a). Immunofluorescence staining revealed increased expression of LC3 in the IL-17-treated group compared to the control group (Fig. 5d).

**Fig. 6:**
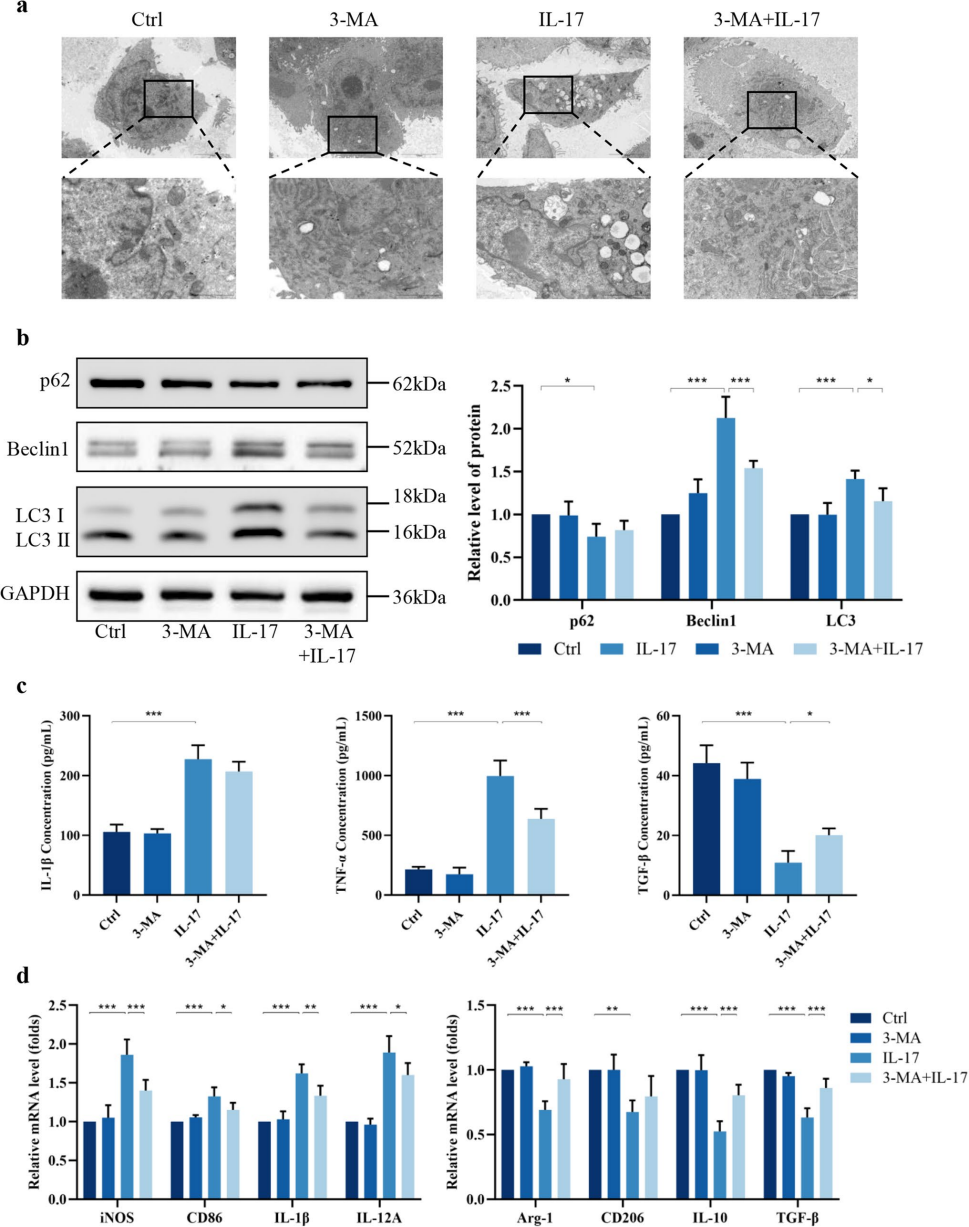
IL-17 is regulating macrophage M1 polarization alteration by activating the autophagic state. **a** 3-MA reduces lysosomal production in macrophages induced by IL-17. Scale bar = 5.0 µm. **b** 3-MA partially inhibits macrophage autophagic activity activated by IL-17 (*n* = 5). **c d** Inhibition of autophagy reduces the IL-17-induced increase in the proportion of M1-macrophages, and the increased secretion of pro-inflammatory cytokines (*n* = 5). ^*^P < 0.05, ^**^P < 0.01, ^***^P < 0.001.

To elucidate whether autophagy plays an active role in IL-17-mediated macrophage M1 polarization, the autophagy inhibitor 3‑MA, a prominent inhibitor that reduces autophagy flux at an early stage, was added. 3-MA alone had no significant effect on the cell membrane structure or morphology but antagonized the IL-17-induced increase in the number of cellular autophagosomes (Fig. 6a). Immunofluorescence staining and WB also revealed that the expression of autophagy-related proteins was significantly downregulated by the addition of 3-MA to IL-17-treated THP-1 cells (Fig. 6b, S5b). This finding suggested that 3-MA inhibits IL-17-induced cellular autophagy.

The ELISA results revealed that the secretion of the proinflammatory cytokines IL-1β and TNF-α was decreased, while the secretion of the inhibitory cytokine TGF-β was increased by 3-MA pretreatment compared to no treatment in the cell supernatants (Fig. 6c). The expression of genes related to M1 and M2 macrophage markers was examined by RT‑qPCR. The addition of 3-MA to IL-17-treated THP-1 cells significantly decreased the expression of iNOS, IL-1β, IL-12A, and CD86, and increased the expression of Arg-1, IL-10, and TGF-β (Fig. 6d). The flow cytometry results showed that 3-MA pretreatment reversed the IL-17-induced increase in the MFI of CD80 and CD86 but had no significant effect on CD163 or CD206 (Fig. S5c). These findings indicated that the inhibition of autophagy abrogates IL-17-mediated M1 macrophage polarization. Similarly, in vivo experiments revealed that the autophagy inhibitor 3-MA reduced macrophage M1-related cytokine expression in the valve tissues of RHD model rats (Fig. S6). Together, these findings indicate that IL-17 regulates alterations in macrophage M1 polarization by activating the autophagic state.

### IL-17 stimulates autophagy through the PINK1/Parkin pathway

**Fig. 7:**
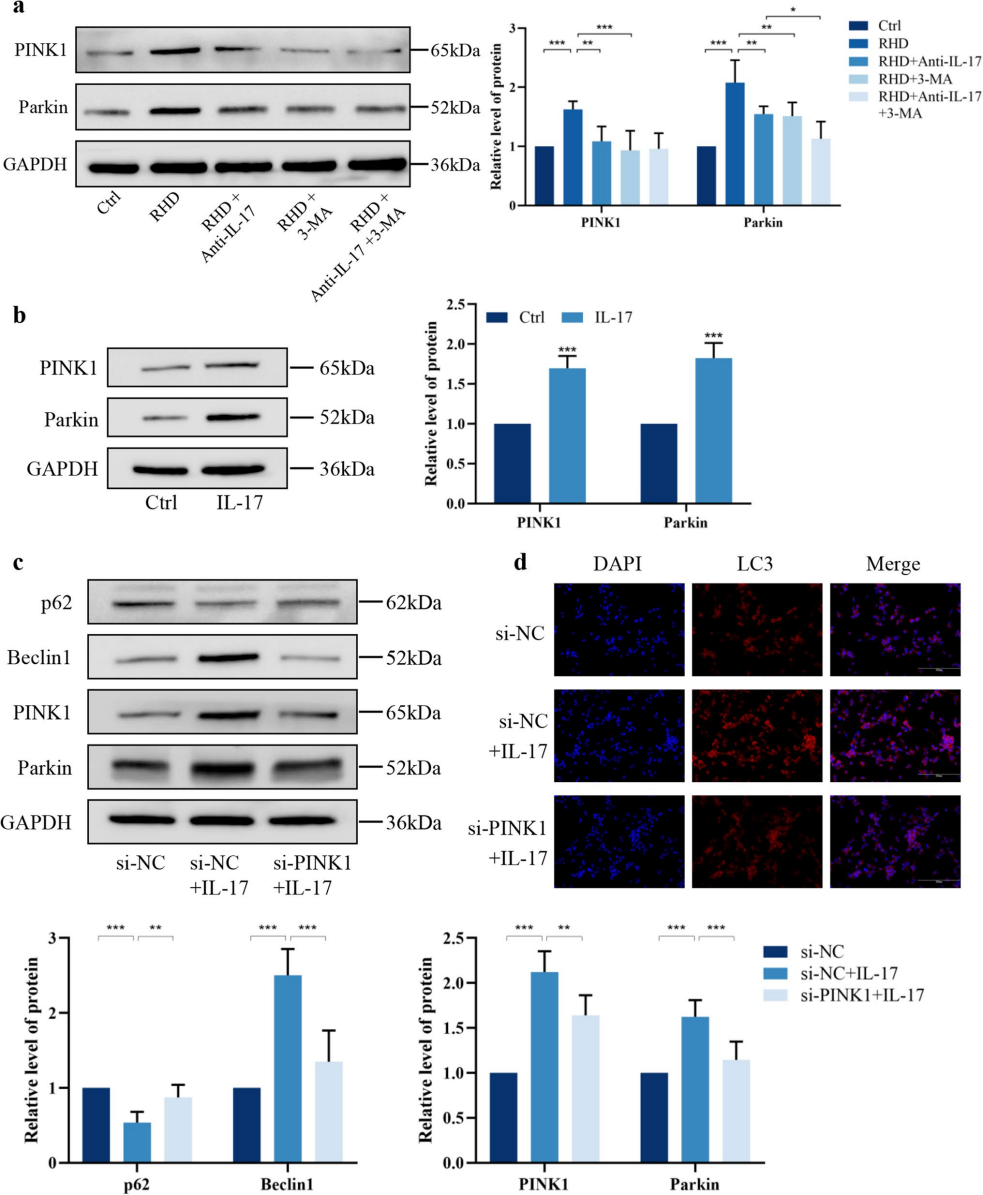
IL-17 stimulates autophagy through activation of the PINK1/Parkin pathway. **a** Anti-IL-17 and 3-MA monotherapy or combination therapy inhibit activation of the PINK1/Parkin autophagic pathway in rat mitral valve tissue (*n* = 6). **b** The protein expression levels of PINK1 and Parkin were up-regulated after IL-17 intervention in macrophages (*n* = 5). Si-PINK1 inhibits activation of the PINK1/Parkin pathway (**c**, *n* = 5) while decreasing the fluorescence intensity of autophagy-related proteins (**d**). ^*^P < 0.05, ^**^P < 0.01, ^***^P < 0.001.

We examined the expression of the key autophagy pathway, PINK1/Parkin, in rat valve tissues and found that compared with those in the control group, the expression of PINK1 and Parkin was increased in the RHD group. Both anti-IL-17 and 3-MA monotherapy also decreased the protein expression of PINK1 and Parkin, and dual blockade significantly reduced Parkin expression more than anti-IL-17 monotherapy (Fig. 7a). This result confirms the involvement of the PINK1/Parkin autophagy pathway is involved in the development of RHD and demonstrates that anti-IL-17 partially reverses the activation of this pathway.

To determine the effects of IL‑17 on the macrophage PINK1/Parkin pathway, we used WB analysis and found that the expression of PINK1 and Parkin was upregulated after the addition of IL‑17 (Fig. 7b). To further investigate whether the activation of autophagy by IL-17 is dependent on the PINK1/Parkin pathway, we silenced PINK1 in THP-1 cells (Fig. S7). PINK1 silencing reduced IL‑17-induced Parkin accumulation in macrophages and partially blocked the effect of IL-17 on autophagy (Fig. 7c, d). Together, these results suggest that IL-17 induces macrophage autophagy in a PINK1/Parkin-dependent manner.

## DISCUSSION

The therapeutic potential of IL-17, a key driver of the immune system, represents a significant breakthrough in immunotherapy for treating inflammatory and autoimmune diseases [32]. Therapies targeting the IL-17 pathway have been approved for the treatment of autoimmunity with positive and effective results [33]. As our understanding of cytokines grows, several clinical trials have begun to explore the efficacy of cytokine-based drugs alone or in combination with other immunotherapies [34, 35]. An in-depth understanding of the specificity of these regulators and the interactions between the factors involved will be essential for the rational design of drug targets. Despite RHD being a common autoimmune condition, there have been no major breakthroughs in its pathogenesis, vaccine development, and therapeutic research in recent decades [36]. Heart valve replacement remains the primary effective treatment option, albeit imposing a substantial economic burden. Our previous study revealed a significant increase in IL-17 expression in valve tissues and serum of RHD patients and model rats. Inhibiting IL-17 production by intervening with the upstream regulatory molecule miR-155 effectively attenuated the inflammatory response in RHD rat valves, indicating that sustained IL-17 activation plays a crucial role in regulating inflammatory injury development in RHD valves [28, 29]. Limited information exists on the cellular roles and molecular regulatory mechanisms of IL-17 in RHD development. Our in vivo results showed that anti-IL-17 treatment attenuated inflammatory damage and reduced EndMT in valves within our well-established RHD rat model, providing supportive evidence for IL-17-targeted therapy in RHD.

The immune system protects the body from internal and external damage by promoting inflammation, with macrophages playing a crucial role in the initiation and resolution of the inflammatory process [37]. Alterations in macrophage phenotype and function can lead to maladaptive repair, resulting in chronic inflammation and pathological fibrosis [38]. Autophagy is a pathway through which proteins, as well as damaged and senescent organelles, are degraded and recycled. New evidence suggests that macrophage autophagy plays diverse roles in macrophage polarization, chronic inflammation, and organ fibrosis due to the high heterogeneity of macrophages in different organs [39]. Recent studies have shown that macrophage autophagy profoundly affects immunity by directly modulating the ability of immune cells to respond to multiple stimuli, indicating that macrophage autophagy may underlie inflammatory disease states and promote increased innate immune activation [40]. However, the specific mechanism by which RHD valve injury is regulated by macrophage autophagy/polarization has not been well established. Our study results using an in vivo RHD model showed increased expression levels of markers related to the macrophage PINK1/Parkin autophagy pathway and macrophage polarization to the M1 phenotype. Treatment with anti-IL-17 effectively reduced macrophage autophagy levels and partially reversed macrophage polarization. These results suggest a potential association between RHD valves and macrophage autophagy/polarization, IL-17 expression, inflammatory injury, and EndMT.

Notably, IL-17 has been shown to play a key role not only in the immune response by inducing macrophage autophagy [41], but also in mediating different polarization states of macrophages [30]. Our current study revealed that treatment of PMA-differentiated THP-1 cells with IL-17 increased the expression of PINK1, Parkin, Beclin1, and LC3, M1 markers, and cytokines, while decreasing the expression or activity of p62 in vitro. The results of the in vivo experiments were consistent with those of the in vitro experiments, and the autophagy inhibitor 3-MA effectively inhibited the PINK1/Parkin autophagy pathway and partially reversed macrophage M1 polarization. This suggests that IL-17-induced macrophage M1 polarization is dependent on the activation of the PINK1/Parkin autophagy pathway. These findings provide further insights into the complex mechanisms involved in RHD pathogenesis and highlight the potential of targeting the IL-17/PINK1/Parkin autophagy pathway as a therapeutic strategy to mitigate inflammatory damage and prevent or reverse EndMT in RHD valves.

EndMT is the process by which endothelial cells lose their endothelial phenotype and acquire mesenchymal characteristics to varying degrees [42]. EndMT imbalance may be the pathological basis of cardiovascular disease, and this imbalance is exacerbated under pathological conditions, forming a vicious cycle. However, because of its reversible properties, EndMT has been recognized as an effective therapeutic target for cardiovascular disease [43]. Heart valve endothelial cells have a unique capacity for EndMT, which is critical during valve development and is thought to be the mechanism by which mature valves are replenished with mesenchymal cells [44]. Previous studies have shown that EndMT is involved in the pathology of RHD-induced mitral valve lesions [6], which is consistent with our current findings. Endothelial cells may be severely affected by a proinflammatory milieu, which has been well described previously. In brief, the activation of proinflammatory signaling pathways (NF-κB and BMPR2) affects a variety of cellular responses, including EndMT [45]. To further characterize the regulatory mechanism of EndMT, we treated HUVECs in vitro for 24 h with CM from macrophages in different activation states and revealed that M1CM decreased the expression of endothelial cell-related markers while increasing the expression of EndMT and fibrosis-related markers. These results provide valuable insights into the mechanisms involved in RHD-induced mitral valve lesions and further support the role of inflammation in driving endothelial cell dysfunction and pathological changes.

The current study has made significant contributions to the field. For the first time, a correlation study between IL-17, macrophage autophagy/polarization, EndMT, and fibrotic injury was performed. The results confirmed that Anti-IL-17 inhibited the activation of the PINK1/Parkin autophagic pathway, reduced the degree of M1-type inflammatory macrophage aggregation, and reversed EndMT-induced fibrotic injury in the valve tissues of RHD rats. This provides strong evidence for targeting IL-17 to treat inflammatory injury in RHD valves. This study also aimed to gain insight into the impact of shifts in macrophage polarity on EndMT progression and its underlying mechanisms. However, this study has some limitations, including the lack of population studies and knockout rat models to further investigate its associations.

## CONCLUSIONS

To summarize, anti-IL-17 treatment can inhibit the activation of macrophage PINK1/Parkin autophagy, reduce the aggregation of M1 inflammatory macrophages, and reverse the damage caused by EndMT in RHD valves. Understanding the role of IL-17 in immunometabolism and its impact on RHD can provide valuable insights for the development of targeted immunotherapies. By targeting IL-17 and related signaling pathways, it may be possible to modulate the immune response and prevent or treat the progression of RHD-induced mitral valve disease.

## Supplementary Information

Below is the link to the electronic supplementary material.

Supplementary file1 (DOCX 1839 KB)

Supplementary file2 (DOC 79 KB)

## Data Availability

No datasets were generated or analysed during the current study.
