# Supplementary material for: Anti-IL-17 Inhibits PINK1/Parkin Autophagy and M1 Macrophage Polarization in Rheumatic Heart Disease
Source: Inflammation. 2024 Jul 8;48(2):870–84. doi: 10.1007/s10753-024-02094-3 (PMC12052801; doi:10.1007/s10753-024-02094-3)
Supplement: Supplementary file 1 — Supplementary file1 (DOCX 1839 KB) [file 10753_2024_2094_MOESM1_ESM.docx]

**Anti-IL-17 inhibits PINK1/Parkin autophagy and M1 macrophage polarization in rheumatic heart disease**

Ling Bai^1 2^, Yuan Li^1 2^, Chuanghong Lu^1 2^, Yiping Yang^3^, Jie Zhang^4^, Zirong Lu^1 2^, Keke Huang^1 2^, Shenglin Xian^1 2^, Xi Yang^2 5^, Na Na^6^, Feng Huang^1 2*^, Zhiyu Zeng ^1 2*^

^1^Department of Cardiology, The First Affiliated Hospital of Guangxi Medical University, Guangxi, China

^2^Guangxi Key Laboratory Base of Precision Medicine in Cardio-cerebrovascular Diseases Control and Prevention, Guangxi Clinical Research Center for Cardio-cerebrovascular Diseases, Guangxi, China

^3^Department of Research, Guangxi Medical University Cancer Hospital, Guangxi, China

^4^Emergency Office, Nanning Center for Disease Control and Prevention, Guangxi, China

^5^Department of endocrinology, The First Affiliated Hospital of Guangxi Medical University, Guangxi, China

^6^Department of Neuroscience, The Scripps Research Institute, La Jolla, USA

Ling Bai, Yuan Li, and Chuanghong Lu contributed equally to this work.

*Correspondence to: ZhiYu Zeng, MD, Department of Cardiology, the First Affiliated Hospital of Guangxi Medical University, Shuang Yong Road 6, Nanning 530021, China, Email [zengzhiyu@gxmu.edu.cn](mailto:zengzhiyu@gxmu.edu.cn); or Feng Huang, MD, Department of Cardiology, the First Affiliated Hospital of Guangxi Medical University, Shuang Yong Road 6, Nanning 530021, China, Email [huangfeng@stu.gxmu.edu.cn](mailto:huangfeng@stu.gxmu.edu.cn).


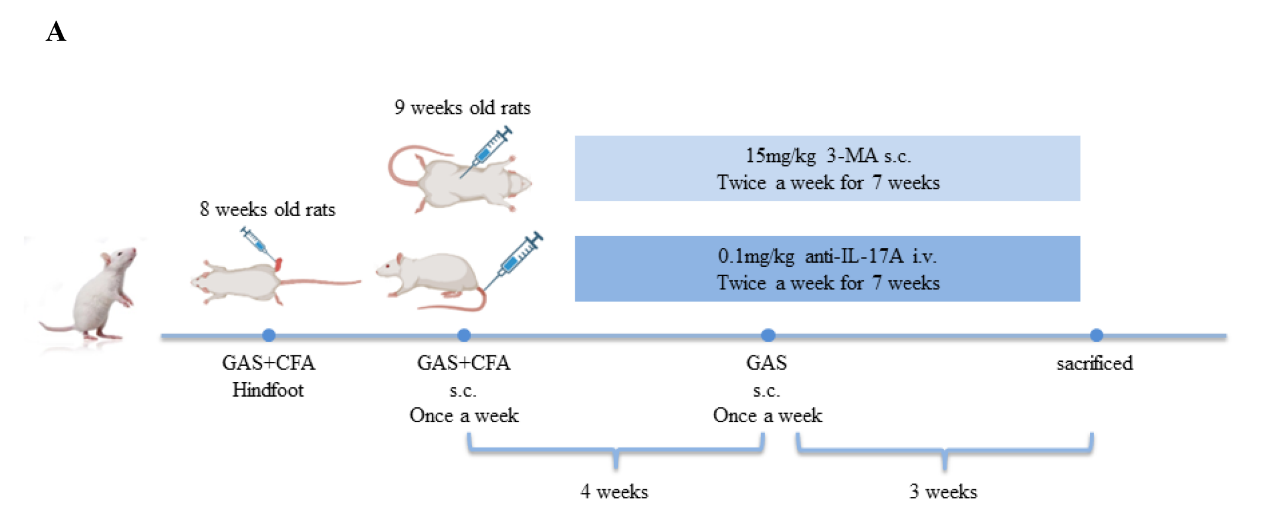
Fig. S1 In vivo experimental study design.


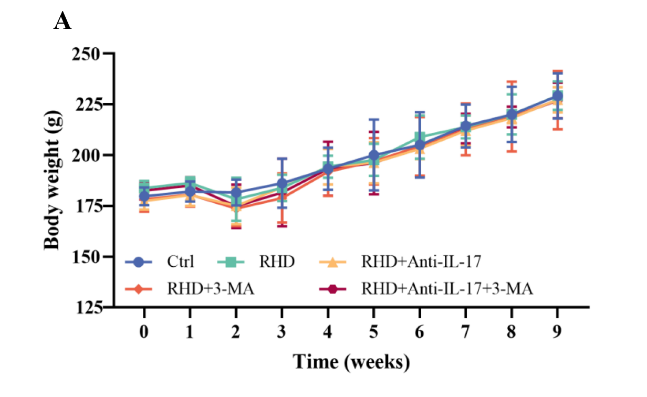
 Fig. S2. Changes in body weight of rats in each group throughout the modeling course (n=6).

Fig. S
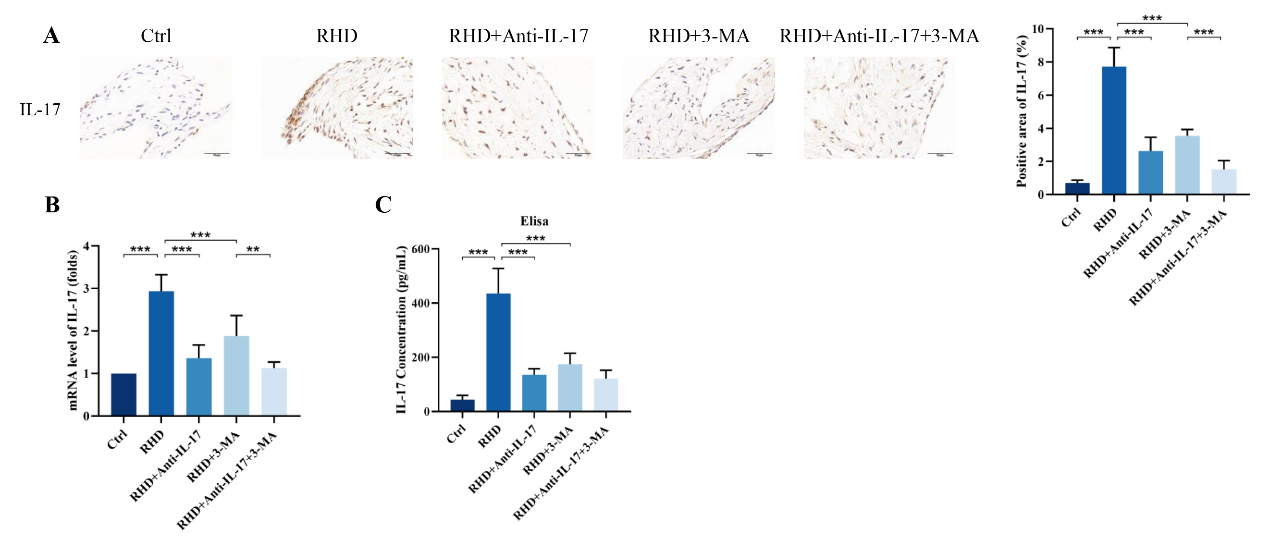
3 Anti-IL-17 and 3-MA monotherapy or combination therapy reduced the level of IL-17 expression in mitral valve tissues of RHD rats. (A) Immunohistochemical staining for IL-17 of rat mitral valve tissues (n=6). Scale bar = 50 µm. The mRNA expression level of IL-17 in mitral valve tissues (B) and serum IL-17 levels (C) were decreased in RHD rats after anti-IL-17 and 3-MA monotherapy or combination therapy (n=6). ^*^*P* < 0.05, ^**^*P* < 0.01, ^***^*P* < 0.001.

Fig. S
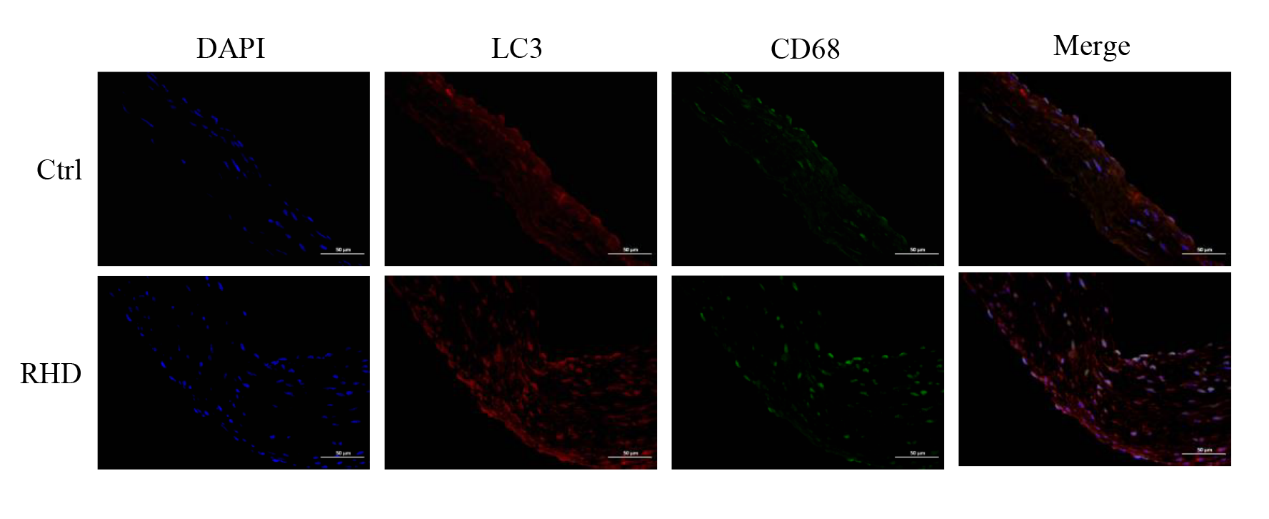
4 Co-localization of LC3 and CD68 in mitral valve tissues of RHD rats. Red fluorescence indicates LC3 protein labeling, green fluorescence indicates CD68 (macrophage marker) protein labeling, and the overlapping position of fluorescent markers indicates yellow fluorescence.

Fig. S
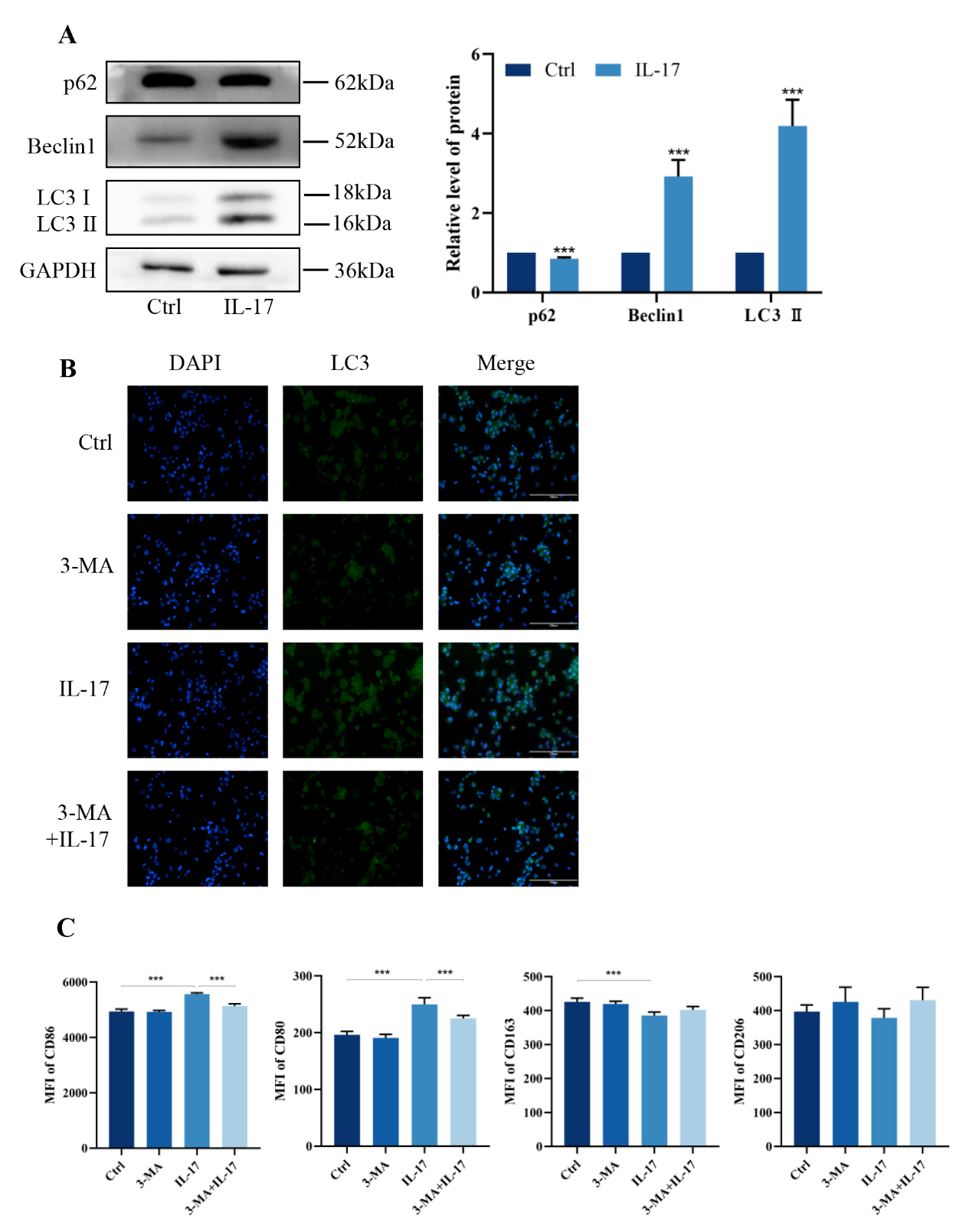
5 3-MA reverses the IL-17-activated macrophage autophagy/polarization pathway. (A) IL-17 elevates the expression level of autophagy-related proteins in THP-1 cells (n=5). (B) 3-MA reduces the fluorescence intensity of IL-17-enhanced autophagy-related proteins. (C) The MFI of M1/M2 macrophage markers after 3-MA pretreatment (n=5). ^*^*P* < 0.05, ^**^*P* < 0.01, ^***^*P* < 0.001.


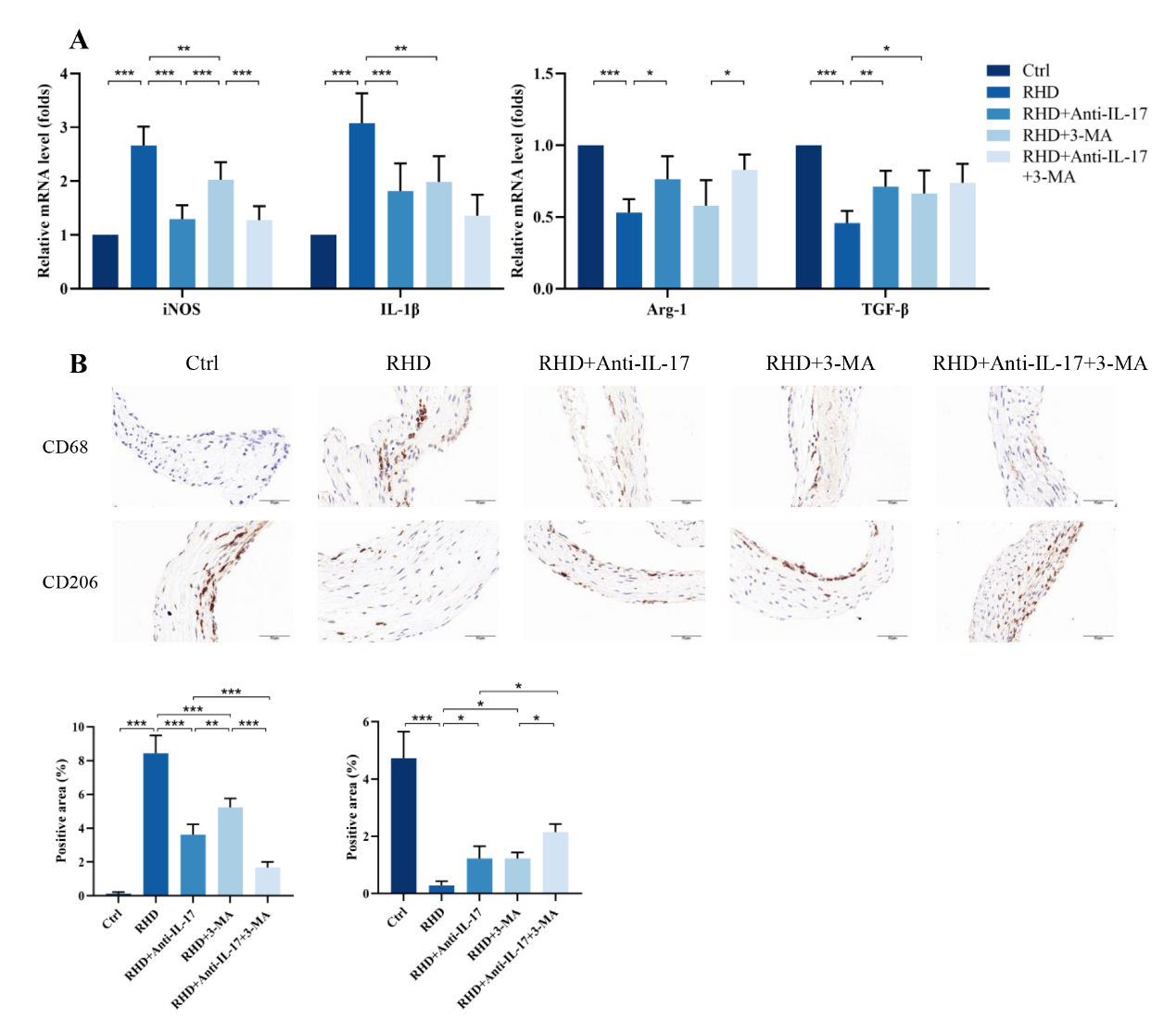
Fig. S6 Inhibition of autophagy attenuates macrophage inflammatory infiltration in RHD valves. (A) RT‑qPCR detected the mRNA expression levels of markers in M1 (iNOS, IL-1β) and M2 (Arg-1, TGF-β) in the mitral valve of rats (n=6). (B) Immunohistochemical staining for CD68 and CD206 (n=6). Scale bar = 50 µm. ^*^*P* < 0.05, ^**^*P* < 0.01, ^***^*P* < 0.001.

Fig. S7
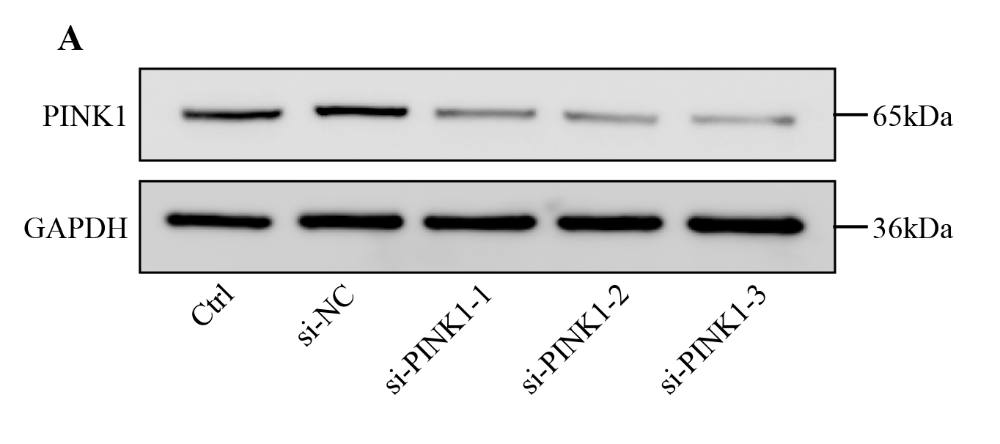
THP-1 cells were transfected with the indicated siRNAs for 48 hours, followed by WB.
