# Supplementary material for: Anti-IL-17 Inhibits PINK1/Parkin Autophagy and M1 Macrophage Polarization in Rheumatic Heart Disease
Source: Inflammation. 2024 Jul 8;48(2):870–84. doi: 10.1007/s10753-024-02094-3 (PMC12052801; doi:10.1007/s10753-024-02094-3)
Supplement: Supplementary file 2 — Supplementary file2 (DOC 79 KB) [file 10753_2024_2094_MOESM2_ESM.doc]

**Anti-IL-17 inhibits PINK1/Parkin autophagy and M1 macrophage polarization in rheumatic heart disease**

Ling Bai^1 2^, Yuan Li^1 2^, Chuanghong Lu^1 2^, Yiping Yang^3^, Jie Zhang^4^, Zirong Lu^1 2^, Keke Huang^1 2^, Shenglin Xian^1 2^, Xi Yang^2 5^, Na Na^6^, Feng Huang^1 2*^, Zhiyu Zeng ^1 2*^

^1^Department of Cardiology, The First Affiliated Hospital of Guangxi Medical University, Guangxi, China

^2^Guangxi Key Laboratory Base of Precision Medicine in Cardio-cerebrovascular Diseases Control and Prevention, Guangxi Clinical Research Center for Cardio-cerebrovascular Diseases, Guangxi, China

^3^Department of Research, Guangxi Medical University Cancer Hospital, Guangxi, China

^4^Emergency Office, Nanning Center for Disease Control and Prevention, Guangxi, China

^5^Department of endocrinology, The First Affiliated Hospital of Guangxi Medical University, Guangxi, China

^6^Department of Neuroscience, The Scripps Research Institute, La Jolla, USA

Ling Bai, Yuan Li, and Chuanghong Lu contributed equally to this work.

*Correspondence to: ZhiYu Zeng, MD, Department of Cardiology, the First Affiliated Hospital of Guangxi Medical University, Shuang Yong Road 6, Nanning 530021, China, Email [zengzhiyu@gxmu.edu.cn](mailto:zengzhiyu@gxmu.edu.cn); or Feng Huang, MD, Department of Cardiology, the First Affiliated Hospital of Guangxi Medical University, Shuang Yong Road 6, Nanning 530021, China, Email [huangfeng@stu.gxmu.edu.cn](mailto:huangfeng@stu.gxmu.edu.cn).

Supplement Table 1. Sequences of PINK1 siRNA.

| PINK1-si1 | Coding strand: | GCUGGAGGAGUAUCUGAUATT |
| --- | --- | --- |
|  | Template strand: | UAUCAGAUACUCCUCCAGCTT |
| PINK1-si2 | Coding strand: | CCUCGUUAUGAAGAACUAUTT |
|  | Template strand: | AUAGUUCUUCAUAACGAGGTT |
| PINK1-si3 | Coding strand: | CCCUGAAGAAUCUGAAGUUTT |
|  | Template strand: | AACUUCAGAUUCUUCAGGGTT |
| NC | Coding strand: | UUCUCCGAACGUGUCACGUTT |
|  | Template strand: | ACGUGACACGUUCGGAGAATT |

Supplement Table 2. Sequences of primers used in RT‑qPCR.

| Gene | Rat | Primer sequence, 5'-3' |
| --- | --- | --- |
| IL-17 | Forward: | TGCCTGATGCTGTTGCTGCTAC |
|  | Reverse: | GGTGAAGTGGAACGGTTGAGGTAG |
| α-SMA | Forward: | GCGTGGCTATTCCTTCGTGACTAC |
|  | Reverse: | CCATCAGGCAGTTCGTAGCTCTTC |
| FSP1 | Forward: | TGGGGAGAAGGACAGACGAAGC |
|  | Reverse: | TGGCAATGCAGGACAGGAAGAC |
| COL1A1 | Forward: | TGTTGGTCCTGCTGGCAAGAATG |
|  | Reverse: | GTCACCTTGTTCGCCTGTCTCAC |
| COL3A1 | Forward: | ACTTCTGGTCCTCCTGGTCTGC |
|  | Reverse: | CGCCTGGCTCACCCTTTTCAC |
| iNOS | Forward: | TCTTGGAGCGAGTTGTGGATTGTTC |
|  | Reverse: | AGTGATGTCCAGGAAGTAGGTGAGG |
| IL-1β | Forward: | AATCTCACAGCAGCATCTCGACAAG |
|  | Reverse: | TCCACGGGCAAGACATAGGTAGC |
| IL-12A | Forward: | TTTGATGATGACCCTGTGCCTTGG |
|  | Reverse: | TCTGCTGATGATTGTGGCTCTGAAG |
| CD86 | Forward: | GCTGTCTCTTTCTGCTGGTCGTC |
|  | Reverse: | CTCACAAGTCTTTCTGCTGGGTCTG |
| TNF-α | Forward: | GCGTGTTCATCCGTTCTCTAC |
|  | Reverse: | GTCTCGTGTGTTTCTGAGCAT |
| Arg-1 | Forward: | CGGCAGTGGCGTTGACCTTG |
|  | Reverse: | GTTCTGTTCGGTTTGCTGTGATGC |
| TGF-β | Forward: | GACCGCAACAACGCAATCTATGAC |
|  | Reverse: | CTGGCACTGCTTCCCGAATGTC |
| β-Actin | Forward: | GGAGATTACTGCCCTGGCTCCTA |
|  | Reverse: | GACTCATCGTACTCCTGCTTGCTG |
| Gene | Human | Primer sequence, 5'-3' |
| iNOS | Forward: | GACTTTCCAAGACACACTTCAC |
|  | Reverse: | TTCGATAGCTTGAGGTAGAAGC |
| IL-1β | Forward: | GCCAGTGAAATGATGGCTTATT |
|  | Reverse: | AGGAGCACTTCATCTGTTTAGG |
| IL-12A | Forward: | CCTTGCACTTCTGAAGAGATTG |
|  | Reverse: | GGTCTCTCTGGAATTTAGGCAA |
| CD86 | Forward: | TGCTCATCTATACACGGTTACC |
|  | Reverse: | TGCATAACACCATCATACTCGA |
| TNF-α | Forward: | ATGACAGACAGAGAGGACAGGAACC |
|  | Reverse: | GGAGGGAGGGAGAGAGGGAGAG |
| Arg-1 | Forward: | GGACCTGCCCTTTGCTGACATC |
|  | Reverse: | TCTTCTTGACTTCTGCCACCTTGC |
| IL-10 | Forward: | GTTGTTAAAGGAGTCCTTGCTG |
|  | Reverse: | TTCACAGGGAAGAAATCGATGA |
| CD206 | Forward: | GACGTGGCTGTGGATAAATAAC |
|  | Reverse: | CAGAAGACGCATGTAAAGCTAC |
| TGF-β | Forward: | CTGTACATTGACTTCCGCAAG |
|  | Reverse: | TGTCCAGGCTCCAAATGTAG |
| α-SMA | Forward: | CTTCGTTACTACTGCTGAGCGTGAG |
|  | Reverse: | CCCATCAGGCAACTCGTAACTCTTC |
| Vimentin | Forward: | CCTTCGTGAATACCAAGACCTGCTC |
|  | Reverse: | CTACCTGCTCTCCTCGCCTTCC |
| FSP1 | Forward: | CGGGCAAAGAGGGTGACAAGTTC |
|  | Reverse: | TTGTCCCTGTTGCTGTCCAAGTTG |
| COL1A1 | Forward: | TGATCGTGGTGAGACTGGTCCTG |
|  | Reverse: | CTTTATGCCTCTGTCGCCCTGTTC |
| COL3A1 | Forward: | CTCAGGGTGTCAAGGGTGAAAGTG |
|  | Reverse: | TGTACCAGCCAGACCAGGAAGAC |
| GAPDH | Forward: | CAGGAGGCATTGCTGATGAT |
|  | Reverse: | GAAGGCTGGGGCTCATTT |
